# The chromosome analysis of the miscarriage tissue. Miscarried embryo/fetal crown rump length (CRL) measurement: A practical use

**DOI:** 10.1371/journal.pone.0178113

**Published:** 2017-06-12

**Authors:** Silvia D’ippolito, Nicoletta Di Simone, Daniela Orteschi, Maria Grazia Pomponi, Maurizio Genuardi, Leuconoe Grazia Sisti, Roberta Castellani, Esther Diana Rossi, Giovanni Scambia, Marcella Zollino

**Affiliations:** 1 Department of Obstetrics and Gynecology, Università Cattolica Del Sacro Cuore, Fondazione Policlinico A. Gemelli, Rome, Italy; 2 Institute of Genomic Medicine, Università Cattolica Del Sacro Cuore, Fondazione Policlinico A. Gemelli, Rome, Italy; 3 Institute of Public Health, Università Cattolica Del Sacro Cuore, Rome, Italy; 4 Division of Anatomic Pathology and Histology, Università Cattolica Del Sacro Cuore, Fondazione Policlinico A. Gemelli, Rome, Italy; CHA University, REPUBLIC OF KOREA

## Abstract

**Objective:**

To investigate whether miscarried embryo/fetal crown rump length (CRL) measurement may yield a practical application for predicting a conclusive result at the cytogenetic analysis of miscarriage tissue. Our study might help in improving the cytogenetic method, the results of which may be affected by maternal cell contamination (MCC). In particular, we aimed at establishing whether the miscarried embryo/fetal CRL measurement shows accuracy in predicting the possibility of MCC and the scan cut-off value useful to this purpose and, as a result, suggest a multi-step procedure for the genetic ascertainment.

**Methods:**

Women experiencing at least two miscarriages of less than 20 weeks size at the Pregnancy Loss Unit at Fondazione Policlinico A. Gemelli underwent a scan before surgery. The CRL value was recorded. After the dilatation and courettage (D&C) procedure, miscarriage tissue was processed through the proposed multi-step procedure before performing oligo-nucleotide-based and SNP (single nucleotide polymorphisms)-based comparative genomic hybridization (CGH+SNP) microarray analysis.

**Results:**

63 women and 63 miscarriages met the criteria. By using the Receiving Operator Characteristic (ROC) curves, CRL showed an AUC of 0.816 (95%CI:0.703–0.928,p<0.001). A CRL≥24.5 mm cut-off value showed a higher positive likelihood ratio (5.27) but, conversely, a higher negative likelihood ratio (0.64) in predicting the possibility of MCC. Microarray analysis was successful in the totality of cases in which the embryo/fetal origin of miscarriage tissues was proven.

**Conclusions:**

The 24.5 mm CRL value emerges as the most suitable cut-off enabling the identification of cases in which the embryo-fetal component can be isolated in the absence of MCC and the chromosomal array provide informative results.

## Introduction

Miscarriage, defined as the pregnancy loss before 20 weeks of gestation, represents a frequent obstetrical event affecting up to 15% of clinically diagnosed pregnancies, yet a failure in the reproductive history of couples hoping for children. [1,2] It is estimated that chromosomal abnormalities, in the majority of cases aneuploidies, account for 50–70% of miscarriages of less than 10 week’ gestation in the general reproductive population [3–9]. In the presence of recurrent pregnancy loss (RPL), it is important to distinguish between parental genetic abnormalities and embryo chromosomal abnormalities. Parental genetic abnormalities contribute for 3 to 5% of RPL cases which is ten times higher than the background population [10–13]. Miscarried embryo chromosomal abnormalities are mostly *de novo* in origin. Nevertheless, in specific cases, chromosomal characterization of miscarriage tissues can identify familial chromosomal rearrangements that may predispose couples to RPL [1]. Even in those cases of *de novo* chromosomal abnormalities, being aware of such information may provide couples with an explanation for the loss. A recent survey on public perceptions of miscarriage reported that up to 75% of all respondents strongly wished to know the cause of their miscarriage, even if no intervention could have prevented it from occurring. In addition, of those participants who experienced a miscarriage 47% felt guilty, 41% reported feeling that they had done something wrong, 41% felt left on their own, and 28% felt ashamed [14]. Even if studies reporting the psychological benefit deriving from cytogenetic testing are still scant [15], this survey suggests that identifying a potential cause of the miscarriage may influence patients' psychological and emotional responses, and possibly help in subsequent pregnancies.

Extant research indicates that the potentials of the standard cytogenetic analysis are limited by different aspects: i) presence of dividing cells; ii) culture failure; iii) overgrowth of maternal cells; iv) microbial contamination; v) poor chromosome morphology [5,16,17]. To overcome these limitations, scientists have started conducting genetic chromosomal microarray testing [1,6,16]. This test allows to perform chromosome analysis on DNA extracted from direct fetal samples without the need for live dividing cells and it can detect quantitative chromosomal abnormalities significantly below the resolution of about 10 megabases (Mb) of the conventional karyotyping [18–20]. Given the small sample size of studies describing microarray testing in pregnancy loss, the American College of Obstetricians and Gynecologists (ACOG) still does not recommend to use it routinely in the RPL standard work-up [21]. In addition, the costs and the percentage of inconclusive results—mainly influenced by maternal cell contamination (MCC) limit its use on large-scale.

MCC occurs when maternal/decidual cells predominantly grow in comparison to embryo/fetal cells [16,22–24] and may be due to both lack of separating the miscarriage tissue from the maternal decidua before culturing and the poor ability of non-euploid embryo/fetal cells to grow, enabling the overgrowth of maternal cells [2,16,22–26]. Throughout the years, different techniques aiming at reducing the rate of MCC have been proposed [24–26]. However, such procedures are limited by a high variability and by their operator-dependent nature. In addition, to our knowledge, scholarly effort has overlooked the possibility of predicting the presence of uninformative results linked to MCC in clinical practice without performing all the steps of the procedure. In this context, the aim of our study is to investigate whether the miscarried embryo/fetal CRL measurement—obtained by using two-dimensional ultrasound—can accurately support the identification of those miscarriage tissues in which the embryo/fetal component can be isolated in the absence of MCC and, therefore, the cytogenetic investigation may provide informative results. Finally, we aimed at establishing a CRL cut-off at which the cytogenetic investigation can generate conclusive result.

## Materials and methods

### Patients

Participants provided a written consent to the anonymous use of their data for research purposes. The protocol was approved by the ethics committee of Fondazione Policlinico A. Gemelli, Università Cattolica del Sacro Cuore in Rome, Italy. The study population included women with miscarriage clinically documented by ultrasonography, attending at our Pregnancy loss Unit (in the period January-June 2016) before undergoing elective dilatation and courettage procedure (D&C). Participation included for all participants a maternal interview, obstetrical ultrasound scan evaluation, chart abstraction, and miscarriage tissue pathological examination. Inclusion criteria were the following: singleton pregnancy; ≥2 miscarriages. The diagnosis of miscarriage was conducted according to international guidelines, based on embryo with a CRL ≥7 mm and no heartbeat [27]. Women were excluded from the study in the presence of one or more of the following criteria: multiple pregnancy; scan suspect of molar pregnancy; diagnosis of miscarriage based on the presence of an empty gestational sac of mean diameter ≥25 mm. Miscarriage specimens were obtained during the D&C procedure. A tube of blood from participating women was collected.

### Genetic counselling

The clinical geneticist offered specific counselling before the genetic test. Participants were informed of the possible (*i*) unsuccessful outcome of the chromosomal microarray analysis due to MCC; (*ii*) presence of a result with quantitative chromosome changes of uncertain significance; (*iii*) presence of medically actionable incidental findings. All women were informed about the result of the procedure.

### Ultrasonography examination

Before surgery, all women underwent scan for the CRL measurement. All scans were performed by one operator who had accumulated at least 3years of experience in the undertaking of obstetrical ultrasound by relying on Esaote MyLab70 machine (Esaote SpA, Florence, Italy). Transvaginal approach by using 9MHz frequency probe was used. CRL measurements were obtained by the two-dimensional ultrasound (2DUS) on the midsagittal plane.

### Pathological examination

Miscarriage tissue was collected through D&C procedure; no suction was performed to collect the tissue. Subsequently, a macroscopic evaluation of the miscarriage tissue was performed in the gross Department of Anatomic Pathology and Hystology. The fresh specimens were evaluated by one pathologist (EDR) in order to isolate the embryo/fetal component in the miscarriage tissue. The criteria for a macroscopic identification of the embryo/fetal component required: a more consistent pattern, a color that tends toward a translucent grey and the embryo/fetal shape. The isolated embryo/fetal component was isolated in an eppendorf tube. The remaining tissue underwent the histological examination. For histological analysis, the miscarriage specimens were fixed in 10% buffered formaldehyde for 20–24 hours, embedded in paraffin and the 5-micron-thick microtomic sections were stained with hematoxylin-eosin.

### Miscarriage tissue processing

After the D&C procedure, the samples were processed following subsequent steps before performing oligo-nucleotide-based and SNP (single nucleotide polymorphisms)-based comparative genomic hybridization (CGH+SNP) microarray analysis. The amount of fetal tissue required for microarray analysis was about 5–10 mg. The pathologist isolated the embryo/fetal component from the whole fresh miscarriage specimen. The embryo/fetal component was isolated from the remaining tissue in those cases where it could be immediately recognized. The isolated miscarried tissue underwent gender assessment by looking at the presence or absence of SRY. Subsequently oligo-CGH+SNP microarray analysis was performed. The remaining tissue was sent for histological examination. When the embryo/fetal component could not be easily identified, the following actions were taken: 1) a further pathological evaluation was performed in order to isolate the embryo/fetal component from the whole miscarriage tissue; the remaining tissue underwent the histological examination; 2) the geneticist performed microsatellite segregation analysis (MSA) on both maternal and likely embryo/fetal DNA of the isolated component; 3) oligo-CGH+SNP microarray analysis was performed on those samples that resulted not to be contaminated (the fetus gender was established by looking at the presence/absence of SRY sequence by Polimerase Chain Reaction).

### Microsatellite analysis (MSA)

When requested, MSA segregation analysis was performed on maternal and miscarriage tissue DNA, by means of at least five polymorphic microsatellites among the following: D6S434, D7S2476, D13S1296, D14S288, D15S153, D20S891, D20S898, D20S842, DXS8051, DXS986, DXS1227, DXS1106, DXS1047 and DXS1060. Paternal DNA was never requested.

### Chromosomal microarray analysis (CMA)

Genomic DNA was extracted from embryo/fetal tissues using QIAamp DNA Blood and Tissue Kit (QIAGEN, Hilden, Germany). Oligonucleotide plus SNP CGH was performed on all samples by using the Baylor College of Medicine Prenatal Research CGH+SNP Microarray kit 4x180K (Agilent Technologies Santa Clara, CA, USA) and in accordance with the manufacturer’s instructions. This platform with 13.4 kb overall median probe spacing and probe enrichment in cytogenetically relevant microdeletion/duplication, pericentromeric and subtelomeric regions, allows the simultaneous detection of copy number and copy neutral aberrations such as absence of heterozygosity, triploidy and tetraploidy. Array images were generated with SureScan scanner and analyzed by Cytogenomics software V 2.7.22.0 (Agilent Technologies Santa Clara, CA, USA).

### Statistical analysis

Median and range were used to describe age and CRL, whereas absolute and relative frequencies for reporting CGH assay. Receiver Operating Characteristic (ROC) curves and Area Under the Curve (AUC) were used to evaluate the accuracy of CRL in identifying cases in which the microarray analysis was successful performed. The result was read according to a minimum AUC value of 0.70 that is conventionally required to define a diagnostic test as accurate. In case of proven accuracy, two cut-off values were identified in order to maximize specificity and the product between specificity and sensitivity. Lastly, positive and negative likelihood ratios were calculated in order to describe the overall accuracy of the cut-off values.

## Results

### Patients

Patients’ characteristics are shown in Table 1. All women underwent two-dimensional ultrasound scans for the miscarried embryo/fetal CRL measurement before the D&C procedure. Overall, 150 women with miscarriage were evaluated. A total of 87 women were excluded from the analysis because of the following conditions: 48 women at their first miscarriage, 36 women with blighted ovum, and 3 women whose scan was suggesting the presence of a possible molar pregnancy. A final population of 63 women with 63 miscarriages met the criteria (S1 Data). They all underwent a dedicated counselling with the clinical geneticist. By analyzing recorded data concerning CRL measurement, we found that the median CRL was 16.5 mm (range 7–95). Among all miscarried embryos, 48/63 (76%) showed a CRL measurement above 10 mm (corresponding to a gestational age of 7 weeks+0 days) [28], 53/63 (84%) showed a CRL measurement below to 33 mm (corresponding to a gestational age of 10 weeks+0 days) [28].

**Table 1:** Characteristics of women in the study.

Characteristics of women (n = 63)	
Age, years (median)	37 (26–45)
BMI, Kg/m^2^	24.03±3.00 (17.96–28.23)
CRL, mm (median)	16.5 (7–95)
Miscarriage <10 weeks of gestation (%)	53 (84%)
Obstetric history, No of cases (%)	
• Primary aborters	43 (68)
• Secondary aborters	20 (32)
✓Two previous miscarriages	28 (44)
✓Three previous miscarriages	18 (29)
✓Four previous miscarriages	11 (18)
✓Five previous miscarriages	6 (9)

**Fig 1:**
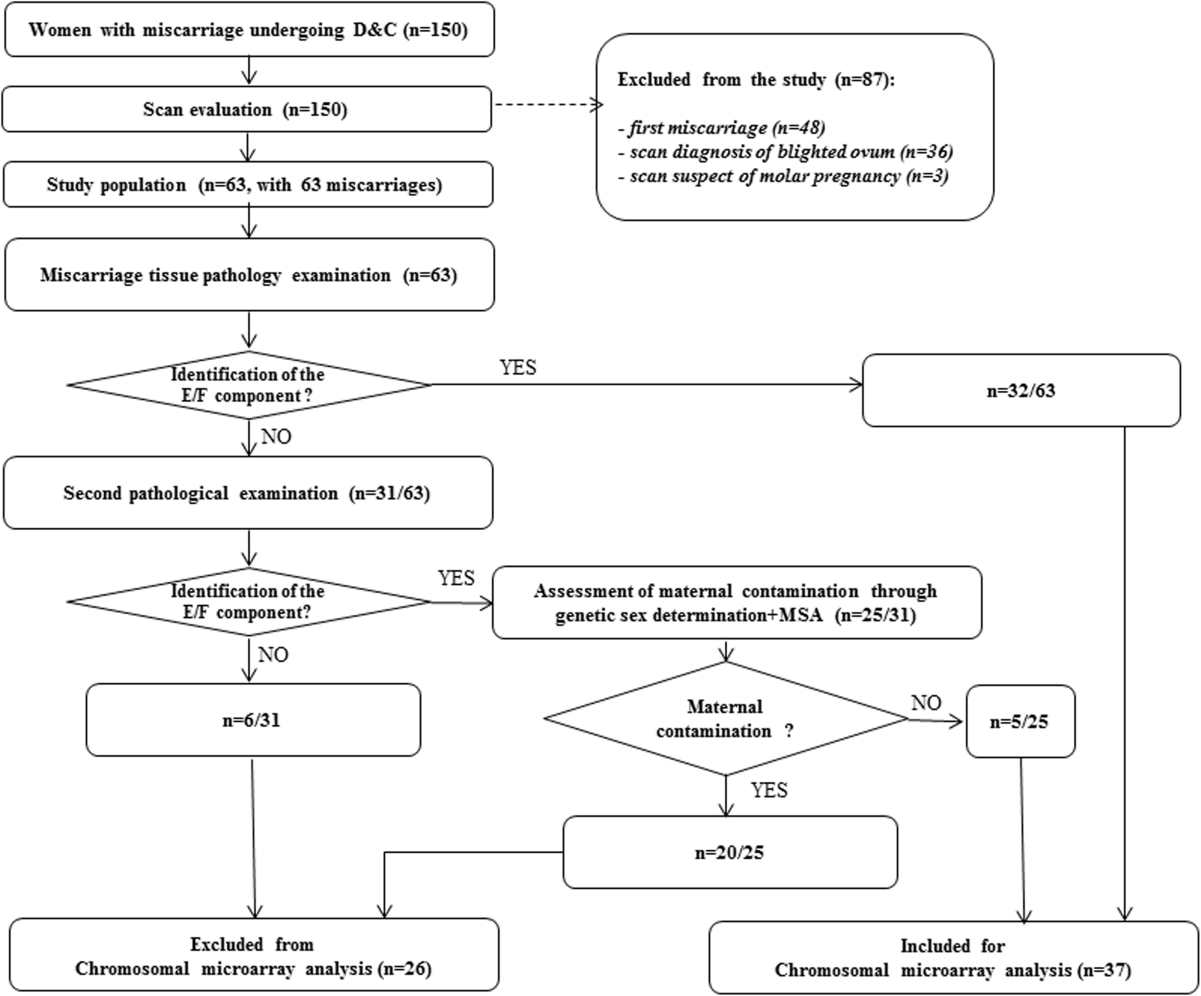
Representative flow-chart of the multistep procedure set-up for the selection of samples for chromosomal microarray analysis. D&C: dilatation and courettage; E/F: embryo/fetal. MSA: microsatellite analysis.

As shown in the flow-chart (Fig 1), in 31 out of 63 miscarriage specimens pathologist could not immediately identify the embryo/fetal component. These specimens showed an average CRL of 15.7 mm (corresponding to a gestational age of 8 weeks + 0 days) [28]. After a further pathological evaluation, the pathologist was able to recognize the embryo/fetal component in 25 out of the 31 miscarriages. To evaluate the presence of MCC, these 25 cases were sent out for genetic sex determination and MSA. MCC was found in 20 miscarriages out of 25. The remaining 5 specimens without MCC were, therefore, sent for chromosomal microarray analysis. The embryo/fetal component was detected immediately after the D&C procedure in 32 cases out of 63 specimens. These specimens showed an average CRL of 28.5 mm (corresponding to a gestational age of 9 weeks + 4 days) [28]. They were all sent for chromosomal microarray analysis.

### Accuracy of the CRL length value

**Fig 2:**
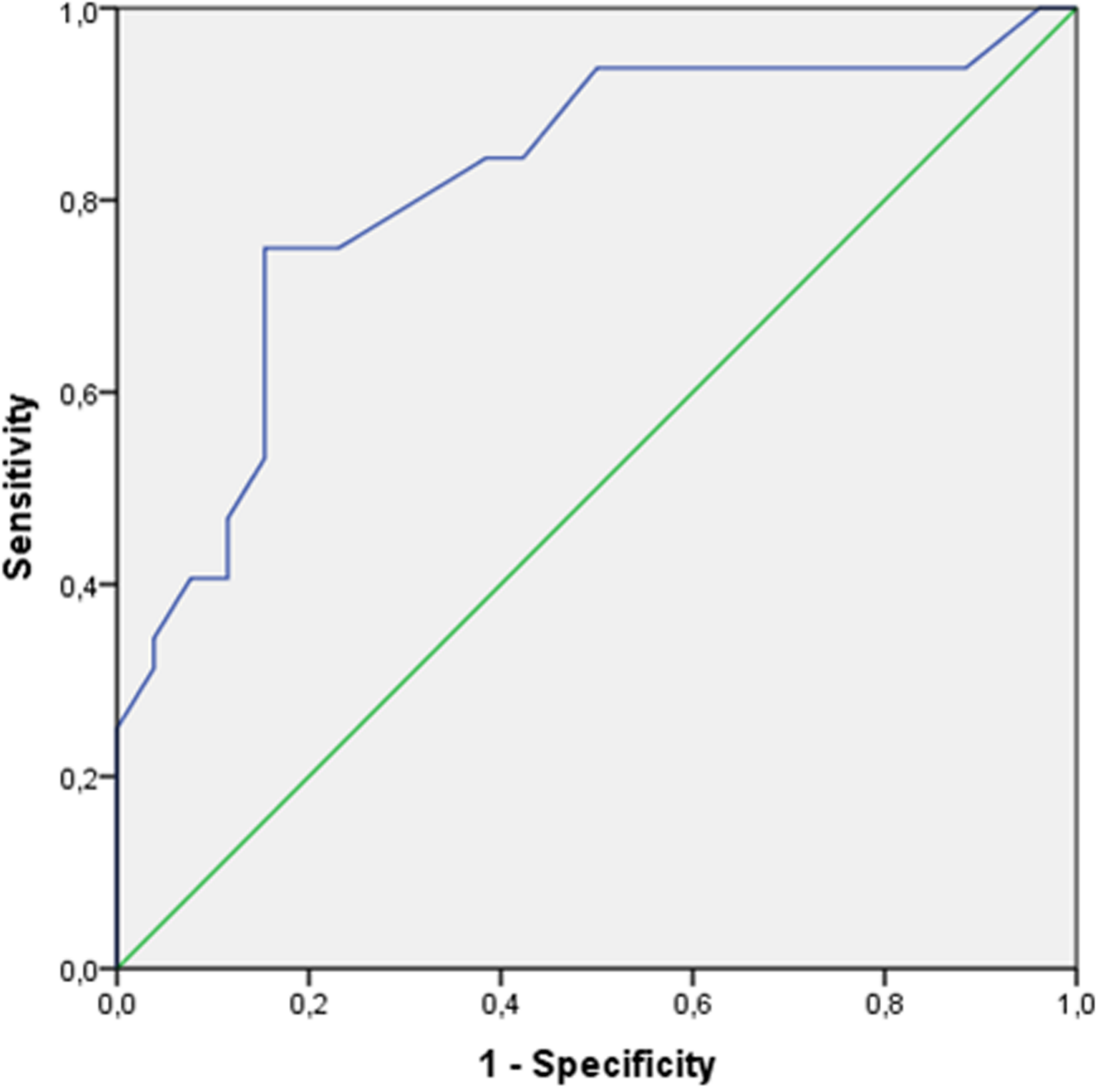
ROC curve showing the accuracy of Crown Rump Length value. The result was read according to a minimum AUC value of 0.70 that is conventionally required to define a diagnostic test as accurate. CRL showed an AUC of 0.816 (95%CI: 0.703–0.928, p < 0.001).

By using the ROC curves, CRL showed an AUC of 0.816 (95%CI: 0.703–0.928, p < 0.001). Therefore the CRL measurement is accurate (Fig 2).

### CRL cut-off values

Two hypothetical cut-off values of CRL were identified: ≥24.5 mm (9 weeks+1 day)^28^, which maximized specificity (92.3%) with a sensitivity of 40.6% and ≥17.5 mm (8 weeks + 2 days) [28], which maximized the product of sensitivity by specificity (specificity: 84.6%; sensitivity: 75%). Positive predictive values were 86.7% and 85.7% respectively whereas negative predictive values were 55.8% and 73.3%. As far as likelihood ratio were concerned, CRL ≥17.5 mm showed a positive likelihood ratio of 4.87 and a negative likelihood ratio of 0.295. CRL ≥24.5 mm showed a higher positive likelihood ratio (5.27) but, conversely, a higher negative likelihood ratio (0.64). In order to identify those cases in which the embryo/fetal component can be isolated in the absence of MCC and, therefore, to be able to rely on a chromosomal array for obtaining informative results, the positive likelihood ratio should be maximized and, subsequently, 24.5 mm may be considered as the most suitable cut-off.

### Chromosomal microarray analysis

As specified above, a total of 37 out 63 miscarriage samples were suitable for chromosomal microarray investigation, after ascertaining embryo/fetal origin of specimens by anatomical direct examination (total 32/63) or by MSA (total 5/25 selected by the pathologist as likely embryo/fetal in origin). Informative results were obtained in the totality of cases, allowing to establish a 100% sensitivity of the oligonucleotide-based and SNP-based array CGH.

Genetic results are shown in Table 2. Chromosome rearrangements were diagnosed in 21 out 37 cases (57%). They consisted of: *a)* autosomal trisomies (7/21): trisomy 15 (2 cases); trisomy 22 (2 cases); trisomy 21 associated with 12p tetrasomy (1 case); trisomy 13 (1 case); trisomy 14 and trisomy 6 (1 case); *b)* monosomy X (4/21); *c)* polyploidy (8/21): 69,XXX (4 cases); 69,XXY (4 cases); *d)* unbalanced translocation: 1/21 (del(1)(q42.3q44) of 13 Mb in size and dup (8)(q22.1q24.3) of 47 Mb in size, segregating from a maternal t(1q;8q) balanced translocation); *e)* structural abnormality: 1/21 (del(X)(p22.33) mat of about 400 kb in size encompassing the *SHOX* gene, in a male fetus), (Table 2). A total of 16/37 (43%) specimens had normal molecular karyotype, which was female in 7 (44%) and male in 9 (56%). A F/M ratio of about 1/1 was observed in cases with normal karyotype, as expected.

**Table 2:** Abnormalities detected by chromosomal microarray analysis. A total of 21 out of 37 (58%).

Anomaly	No of cases	Type
Trisomies	7	Trisomy 15 (No 2)
		Trisomy 22 (No 2)
		Trisomy 21 + tetrasomy 12p (No 1)
		Trisomy 14 + trisomy 6 (No 1)
Monosomies	4	Monosomy X (No 4)
Polyploidies	8	69,XXX (No 4)
		69,XXY (No 4)
Unbalanced translocations	1	Unbalanced t(1q;8q) * causing del(1)(q42.3q44) of 13 Mb and dup(8)(q22.1q24.3) of 47 Mb
Interstitial anomalies	1	Del(X)(p22.33)mat of 400 kb**

*Unbalanced segregation of a balanced t(1q;8q) maternal translocation.

**Detected in a male fetus.

## Discussion

In the present study, we proposed to find a significant value for a clinical parameter, i.e., embryo/fetal CRL, as a predictive marker for directing miscarried products of conception for cytogenetic analysis. In particular, we found that pre-surgery embryo/fetal CRL measurement shows accuracy in identifying those miscarriage samples in which the embryo/fetal component can be isolated in the absence of MCC and we detected 24.5 mm as the cut-off value. The anatomical identification of the fetal component was associated with an average CRL of 28.5 mm. Accordingly, it may be appropriate to conduct the chromosomal microarray analysis only with regard to the miscarriages that meet this criterion. However, a total of 5 out 25 specimens with an average CRL of 15.7 mm, with no obvious anatomical evidence of embryo-fetal components, were fetal in origin, as detected by MSA. Thus, inclusion criteria may reflect attitudes of individual diagnostic units.

In the recent years, increasing number of studies perform cytogenetic analysis of the miscarriage tissue starting from the second miscarriage and even propose this approach as a more cost-effective strategy compared to the evidence-based standard RPL evaluation [6,16,21,29]. In this perspective, these studies suggest that the chromosome evaluation of miscarriage tissue should be carried out as a first step, before deciding whether to proceed with the standard RPL or not [9,30,31]. MCC represents an important limitation to the accuracy of the miscarriage tissue genetic analysis. It is mainly due to the lack of separation of the miscarriage tissue from the maternal decidua and its incidence varies in the literature from twenty-nine to 90% [26,32]. Polymerase chain reaction (PCR) used on first trimester spontaneous abortion specimens demonstrated that at least 30% of 46XX results are due to MCC [26,32,33]. Given the specificity of the probes used for this technique, only errors due to XY fetuses are detectable; as a result, it has been hypothesized that the true error rate of conventional cytogenetics is even higher because of undetected aneuploidies. Accordingly, Lathi et al. through molecular analysis of 1222 miscarriage specimens revealed an overall MCC rate of 22% and even of 59% in the 46XX results [34]. Over the years, to overcome this limit researchers have tried to suggest specific tissue sampling techniques including careful separation of the villous tissue and thorough washing with saline prior to sending missed abortion specimens for the genetic testing [33]. In addition, an explanatory video has been published to illustrate the procedure of separation of miscarriage tissue from the maternal decidua [24]. However, the high dependence of these techniques on the operator ability and on the availability of specific devices used for the uterine evacuation may limit their application. These techniques do not allow to predict the result of the procedure and, therefore, all the steps for the genetic testing must be performed. This may have consequence on the costs of the procedure and on the expectations of frustrated couples at their second miscarriage. For the aims of defining a practical tool that could improve the efficiency of the genetic procedure and the counselling to couples, we intend to find a clinical parameter able to predict the presence of MCC and therefore a conclusive result at the genetic testing. At the time of tissue processing, we considered the selection of the embryo/fetal component in the fresh miscarriage samples a crucial step. Those cases, in which the pathologist detected the embryo/fetal component immediately after the D&C procedure, were directly sent for array analysis. On the contrary, the remaining cases were further processed and then sent out for MCC detection through MSA. As we were moving through these steps, we were able to send for the final chromosome analysis by oligo-CGH+SNP microarray a total of 37 out 63 miscarriages (58.7%). Successful informative results were obtained in all of them. Chromosome anomalies were detected in 57% of cases, consisting of autosomal trisomies (33%), X monosomy (19%), polyploidy (38%), unbalanced translocation (5%) and structural anomaly (5%). Noteworthy, the unbalanced translocation segregated from a maternal balanced translocation: this diagnosis allowed a proper genetic counselling in the family. In line with previous studies, our results support the utility of microarray analysis for miscarriage tissue [35–38]. The present results are comparable to other studies [36,37]. It would be difficult to compare the frequencies of detected abnormalities in our study with the previous published frequencies since the sample size of our study is small and does not allow a similar comparison. At this regard, we recognize that our research did not compare different rates of informative chromosome analysis; instead, we identified a practical tool that could support clinicians, researchers, and maternal-fetal-medicine readership in predicting the likelihood of a failed chromosome test. To this end we decided to retrospectively analyze collected data from patients attending our clinic during a limited period (from January through June 2016). To our knowledge this is the first study investigating any correlation between embryo/fetal anatomical parameters and risk for MCC and/or unsuccessful cytogenetic analysis.

In conclusion, by analyzing the pre-surgery CRL value, we found that CRL is an accurate parameter to identify those miscarriages in which the embryo/fetal component can be isolated in the absence of MCC and we established a scan cut-off (>24.5 mm) that could enable the identification of those cases in which it is justified to enable each step for the chromosomal microarray analysis. Additional research in this direction is urged to verify the recommendations suggested above as well as quantify the potential economic impact of this approach.

## Supporting information

S1 Data Data for statistical analysis. (XLSX)
